# Implication of using cognitive function-related simple questions to stratify the risk of long-term care need: population-based prospective study in Kobe, Japan

**DOI:** 10.1186/s12961-022-00920-4

**Published:** 2022-11-29

**Authors:** Shinsuke Kojima, Takashi Kikuchi, Yasumasa Kakei, Hisatomo Kowa, Yasuji Yamamoto, Hiroyuki Kajita, Tohmi Osaki, Masanori Fukushima, Ryoma Kayano, Yoji Nagai

**Affiliations:** 1 Translational Research Center for Medical Innovation, Foundation for Biomedical Research and Innovation, Kobe, Hyogo Japan; 2 Clinical and Translational Research Center, Kobe University Hospital, Kobe, Hyogo Japan; 3 Division of Cognitive and Psychiatric Rehabilitation, Department of Rehabilitation Science, Kobe University Graduate School of Health Sciences, Kobe, Hyogo Japan; 4 Department of Biosignal Pathophysiology, Kobe University Graduate School of Medicine, Kobe, Hyogo Japan; 5 Medical Center for Student Health, Kobe University, Kobe, Hyogo Japan; 6 Faculty of Rehabilitation, Kobe Gakuin University, Kobe, Hyogo Japan; 7 Department of Psychiatry, Kobe University Graduate School of Medicine, Kobe, Hyogo Japan; 8 Learning Health Society Institute, Nagoya, Aichi Japan; 9 World Health Organization Centre for Health Development, Kobe, Hyogo Japan; 10 Department of Clinical Research Facilitation, Institute for Advancement of Clinical and Translational Science, Kyoto University Hospital, 54 Kawaharacho, Shogoin, Sakyo-Ku, Kyoto, 606-8507 Japan; 11 Division of Translational Science, Kobe University Graduate School of Medicine, Kobe, Hyogo Japan

**Keywords:** Dementia, Frailty, Long-term care, Administrative data, Population-based study

## Abstract

**Background:**

This study investigated how cognitive function-related simple questions can be used to identify older individuals who are at risk of needing long-term care.

**Methods:**

This cohort study was conducted in Kobe city, Japan. In 2015, the municipal office distributed the Kihon Checklist by post, a 25-item questionnaire including three cognitive function-related questions (questions 18, 19, 20) to citizens aged ≥ 70 years. Need certification is routinely done by Kobe city as part of the national Long-Term Care Insurance Act. The answers to the 2015 questionnaire were merged with need certification data between the questionnaire delivery and the end of December 2019.

**Results:**

Of the 77,877 citizens (age: 72.9 ± 2.7 years) who received the questionnaire, 50,154 responded (response rate: 64.4%). During the study period, the cumulative incidence of the need for long-term care was higher in those who did not respond than in those who did (12.5% vs 8.4%; *P* < 0.001). Among those who responded, the incidence of the need for long-term care was progressively greater as the number of negative answers to cognitive function-related questions increased (5.0%, 8.4%, 15.7% and 30.2% at 4 years’ follow-up, for respondents with, respectively, 0, 1, 2 and 3 negative answers). Similarly, when the need certification for long-term care was confined to that accompanied by dementia, the incidence also rose as the number of negative responses to the cognitive function-related questions increased (3.4%, 6.5%, 13.7% and 27.9% for respondents with, respectively, 0, 1, 2 and 3 negative answers). Using multivariate Cox regression analysis, all three cognitive function-related questions were predictive of the need for long-term care, and question 18 (about memory loss) had the highest hazard ratio for predicting the need for long-term care accompanied by dementia.

**Conclusions:**

Use of cognitive function-related simple questions may help identify older adults at risk for needing long-term care, suggesting their potential value for use in administrative and policy approaches aimed at reducing the societal burden of dementia.

**Supplementary Information:**

The online version contains supplementary material available at 10.1186/s12961-022-00920-4.

## Background

In Japan, the proportion of older individuals aged ≥ 65 years was 28.4% in 2019, and it is expected to reach 38.1% in 2060 [1]. Moreover, in 2017 Japan had the highest prevalence of dementia (2.3%) among countries in the Organisation for Economic Co-operation and Development, and the prevalence is projected to reach 3.8% by 2037 [2]. In the face of such an unprecedented super-aged society, the societal burden of elderly care is a pressing challenge for the nation. Specifically, dementia is one of the leading causes of the need for long-term care (LTC), accounting for 24.3% of LTC need certifications in Japan in 2019 [3]. Although significant advances have been made during the past decade in neuroimaging and in understanding clinicopathological correlations and biomarker development, improvements in the treatment have been limited, partly due to the lack of efficient methods for screening and detecting high-risk individuals.

To prepare for the coming super-aged society, the Japanese government implemented a revised initiative in 2006 [4] to reduce the increasing need for LTC, which included the implementation of the Kihon Checklist (KCL) survey [5, 6]. The KCL is a 25-item questionnaire, designed by the Ministry of Health, Labour and Welfare, that aims to identify frail older individuals. Additionally, certification of need for LTC has been in place across the nation since 2000 under the LTC Insurance Act [7]. Under such conditions, we have previously analysed the relationships between responses on the KCL survey and certification data for LTC by merging two kinds of data separately stored in the Kobe municipal office. Through these analyses, we have found a temporal link between negative answers in the KCL—that is, answers that reflect a decline in cognitive function—and the risk for LTC for approximately 180 000 citizens in Kobe [8]. Of note, the need for LTC in citizens with three negative answers was nearly 10 times higher than in those with no negative answers. However, because the analyses were performed on merged data collected for different purposes, the accuracy of the findings needed to be addressed. Indeed, certain missing data in the KCL survey had been coded as negative answers for administrative purposes. Also, the underlying diseases of those with LTC needs could not be addressed, precluding argument on the causal link between negative answers and LTC need.

Thus, this study aims to strengthen and extend the findings of our prior study by prospectively evaluating the relationship between negative answers on the KCL survey and the risk for future LTC need among the older population of Kobe city.

## Methods

### Study design

This study was conducted as part of the “Kobe project for the exploration of newer strategies to reduce the societal burden of dementia”, which was a collaborative research project of Kobe University, the Foundation for Biomedical Research and Innovation, Kobe Gakuin University and WHO. The Kobe municipal office provided strong support for the project. The rationale, overall design, outline of the studies and final goals of the project have been described elsewhere [9].

This is one of four studies included in the above Kobe project, and is a cohort study conducted with the administrative services of Kobe city to examine the relationships between cognitive decline, as evidenced by negative answers in the KCL, and the incidence of need for LTC in the 4 years after the survey.

### Study population

Based on population statistics, Kobe is the sixth largest city in Japan, with 1,500,000 residents; the potential respondents were citizens aged ≥ 70 years. From May to July 2015, the Kobe city municipal office distributed by post the KCL questionnaire to residents in this age group whose age was an even number (e.g. 70, 72, 74), representing roughly half of the target population.

The KCL questionnaire was delivered to 77,879 citizens who had not already been certified as needing LTC. However, two potential respondents were deceased, so the study sample comprised 77,877 residents with a mean age of 72.9 ± 2.7 years, and 46.0% of respondents were men.

### Baseline evaluation

The KCL evaluates respondents’ ability to care for themselves in order to identify frail older adults [5, 6]. The simple questionnaire was developed by the Ministry of Health, Labour and Welfare. The 25 items assess respondents’ abilities in seven domains: activities of daily living (ADL) (questions 1–5), motor activities (6–10), nutrition (11 and 12), oral function (13–15), frequency of going out (16 and 17), cognitive function (18–20) and mental status (21–25). The three questions that reflect cognitive function focus on memory loss (question 18), executive function (19) and temporal orientation (20). Question 18 asks respondents, “Do your family or your friends point out your memory loss?”; question 19 asks, “Do you make a call by looking up phone numbers?”; question 20 asks, “Do you find yourself not knowing today’s date?” Additionally, although the 26th question (a 5-point rating scale of subjective general health status, thereafter proposed by the Ministry of Health, Labour and Welfare) was included in the KCL delivered by Kobe city, it was not considered in this study, in order to be consistent with the original definition [6].

### Outcome evaluation

Under the national LTC Insurance Act, applications for LTC need certification are submitted from people who wish to be certified to receive care services at lower cost. When the municipal office receives applications from citizens, their health and living conditions are meticulously evaluated according to government guidelines, and certification is based on a predetermined algorithm, with the final judgement made by a publicly authorized committee. When LTC need is certified, the levels of need range from LTC level 1 (least severe) to LTC level 5 (most severe). LTC level 1 is certified when an individual shows instability in walking or standing up and requires some support in their daily life, for example, needing help with bathing; LTC level 5 is certified when comprehensive care is required for all aspects of daily living and this occurs concomitantly with difficulty in communication. Using these data, the primary outcome of this study was the time from completing the survey in 2015 to the occurrence of LTC need certification (LTC level 1 or worse).

During the certification processes, ADL impairment due to dementia is also evaluated and categorized, ranging from independent to grade IV (worst impairment) (Additional file 1). Using these categories, this study defined “LTC need with dementia” to be a need for LTC accompanied by dementia-related ADL impairment at grade IIa or worse, where grade IIa represents the condition in which, despite symptoms or behaviours interfering with daily life or making communication difficult, the individual is independent, although care is required outside the home.

### Data collection and management

In our prior study [8], missing data in the KCL surveillance had been coded as negative answers according to the local practice of the Kobe municipal office, which could have impacted on the risk associated with respective questions. Thus, in the current study, the returned KCL answers were recorded on the database exactly as they were, with missing or indiscernible answers treated as blank data. Data on LTC need certification were extracted from a separate administrative database. Then, the municipal office merged the KCL data with the LTC need certification data to prepare the data set for analysis. After all personal information was removed, the data set was transferred to our study group for statistical analyses.

### Statistical analyses

#### Incidence of need certification

The cumulative incidence of need certification for LTC at 1, 2, 3 and 4 years after the 2015 survey was evaluated using the Kaplan–Meier method and log-rank test, and intergroup differences were compared between those who returned the survey and those who did not. The duration of follow-up was calculated from the date the survey was posted to the date of LTC need certification.

#### Impact of answers to cognitive function-related questions

Based on the number of negative answers to the three questions in the cognitive domain of the KCL (questions 18–20), respondents were divided into four groups: (i) no negative answers, (ii) one negative answer, (iii) two negative answers and (iv) three negative answers. The incidence of LTC was similarly evaluated as above.

#### Exploring the impact of cognitive function-related questions on the risk of LTC need

To explore risk factors for LTC need, multivariate Cox regression analyses were carried out using 27 explanatory variables, comprising age, sex and questions 1–25 in the KCL. To find the minimum adequate model, variables were eliminated in a stepwise fashion from the initial model using Akaike’s information criterion.

Statistical analysis was conducted using SAS version 9.4 (SAS Institute, Cary, NC, USA), and the significance threshold was set at *P* < 0.05.

### Ethical considerations

This study was approved by the WHO Research Ethics Review Committee (Protocol No. 0002899); the Ethics Review Committee of the Foundation for Biomedical Research and Innovation, Kobe, Japan (Approval No. 16-06); and the Ethics Review Committee of the Kobe municipal office (Approval No. 29-2). This study conforms to the provisions of the Declaration of Helsinki, and was conducted in accordance with Japan’s “Ethical guidelines for medical and health research involving human subjects” and, as such, informed consent was not required to analyse anonymized data. Instead, the study outline was posted on the Kobe municipal office website, where protection of personal information and complete anonymity were ensured.

## Results

### Baseline characteristics

Of the 77,877 citizens who received the KCL questionnaire from the municipal office, 50,154 responded and 27,723 did not, corresponding to a response rate of 64.4%.

The percentages of negative answers are listed in Table 1. Question 10, which asks respondents whether they are worried about falling, was the question that most often had a negative answer (37.0% of respondents), whereas question 2, which asks respondents about their ability to buy daily necessities, had the fewest negative responses (2.8%). For the three cognitive function-related questions, the percentages of negative answers were moderate compared with responses to other questions: 9.5% responded negatively to question 18 (about repeating themselves and feeling forgetful), 8.3% responded negatively to question 19 (about looking up phone numbers) and 18.0% responded negatively to question 20 (about knowing the date).

**Table 1:** Percentage of negative answers in the KCL, Kobe, Japan, 2015 (*N* = 50,154)

Question no	Item	Negative answer (%)	Data missing (%)
1	Do you go out by bus or train by yourself?	3.4	0.6
2	Do you go shopping to buy daily necessities by yourself?	2.8	0.5
3	Do you manage your own deposits and savings at the bank?	7.7	0.7
4	Do you sometimes visit your friends?	25.9	1.3
5	Do your family or friends turn to you for advice?	10.8	1.4
6	Do you normally climb stairs without using a handrail or the wall for support?	27.1	1.3
7	Do you normally stand up from a chair without any aids?	9.9	0.9
8	Do you normally walk continuously for 15 minutes?	6.0	0.8
9	Have you experienced a fall in the past year?	16.9	0.8
10	Do you have a fear of falling while walking?	37.0	1.5
11	Have you lost 2 kg or more in the past 6 months?	12.9	3.8
12	Height: cm, weight: kg, BMI: kg/m^2^ If BMI is less than 18.5, this item is scored negatively	7.3	6.9
13	Do you have any difficulties eating tough foods compared to 6 months ago?	20.1	6.9
14	Have you choked on your tea or soup recently?	19.4	0.8
15	Do you often experience having a dry mouth?	22.6	0.8
16	Do you go out at least once a week?	3.5	1.1
17	Do you go out less frequently compared to last year?	17.0	0.8
18	Do your family or your friends point out your memory loss? e.g. “You ask the same question over and over again”.	9.5	1.0
19	Do you make a call by looking up phone numbers?	8.3	1.2
20	Do you find yourself not knowing today’s date?	18.0	0.8
21	In the last 2 weeks have you felt a lack of fulfilment in your daily life?	13.2	1.3
22	In the last 2 weeks have you felt a lack of joy when doing the things you used to enjoy?	9.9	1.6
23	In the last 2 weeks have you felt difficulty in doing what you could do easily before?	25.2	1.3
24	In the last 2 weeks have you felt helpless?	15.1	1.6
25	In the last 2 weeks have you felt tired without a reason?	24.3	2.3
	Average	15.0	1.7

*BMI* body mass index

Among the 25 questions, the proportion of missing data ranged from 0.5% (question 2) to 6.9% (question 12 and 13), with an average of 1.7%.

### Incidence of certification for LTC need

**Fig. 1:**
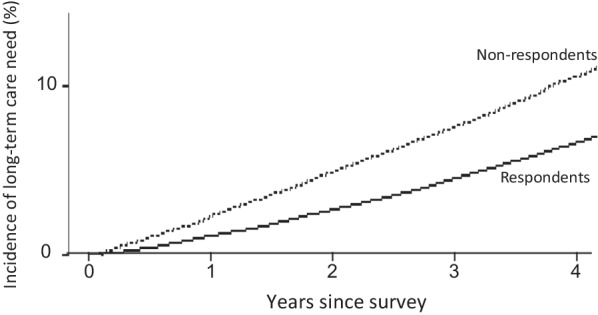
Incidence of LTC need, by response or nonresponse to the KCL survey. The need for LTC was higher among people who did not respond (dashed line) to the questionnaire than among those who did (solid line)

During the follow-up period from May 2015 to December 2019, the incidence of need certification for LTC was 8.4% (4208 of 50 154) for those who returned the KCL, and 12.5% (3,470 of 27 723) among those who did not, implying there was a higher risk for LTC need among those who did not respond (univariate hazard ratio: 1.55, 95% CI: 1.48–1.62, *P* < *0.001*; age- and sex-adjusted hazard ratio: 1.63, 95% CI: 1.55–1.70, *P* < 0.001). Also, at each follow-up year, the incidence of LTC need was consistently higher in those who did not respond than in those who did (2.2 vs 1.1% at year 1; 4.9 vs 2.7% at year 2; 7.6 vs 4.5% at year 3; and 10.6% vs 6.7% at year 4) (Fig. 1).

### Association between negative answers and future need for LTC

**Fig. 2:**
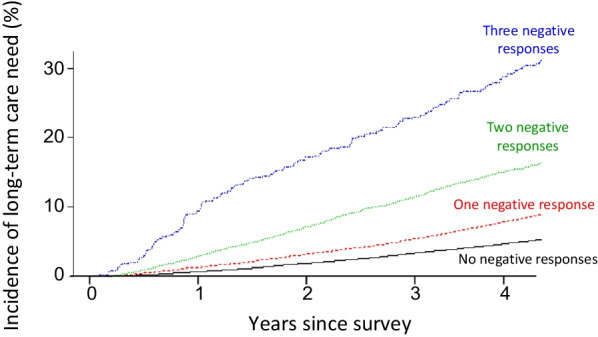
LTC need by number of negative answers to cognitive function-related questions in the KCL. The incidence of need for LTC increases as the number of negative answers to questions in the cognitive domain increases

For the 50,154 respondents, the cumulative incidence of LTC need progressively increased as the number of negative answers increased for the questions in the cognitive domain (Fig. 2). Of note, the incidence of need at the 4-year follow-up was particularly high as the number of cognitively unfavourable answers in the KCL increased (5.0% among respondents with no negative response; 8.4% among those with one negative response; 15.7% among those with two; and 30.2% among those with three).

**Fig. 3:**
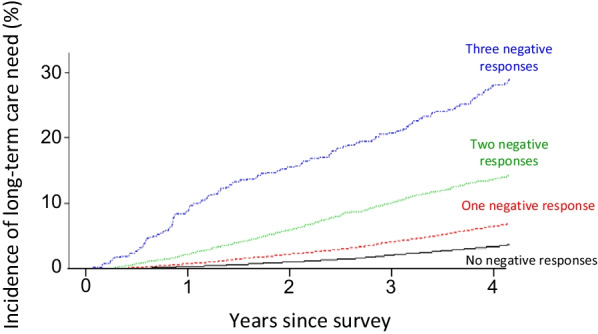
LTC need with dementia by number of negative answers to cognitive function-related questions in the KCL. The incidence of need for LTC with dementia increases as the number of negative answers to questions in the cognitive domain increases

Also, among the 4,208 respondents who were certified to have a need for LTC, 2,821 (67.0%) were categorized as “LTC need with dementia”. When the analysis was confined to the group “LTC need with dementia”, again the incidence of need was higher as the number of negative answers increased to questions in the cognitive domain (3.4% for those with no negative answers; 6.5% for those with one negative answer; 13.7% for those with two; and 27.9% for those with three) (Fig. 3).

### Using answers to conduct a risk assessment of future needs for LTC

A multivariate Cox regression analysis was used to evaluate the impact of answers to the survey on the risk for LTC (Table 2). Using stepwise elimination from the initial model, many of the questions, including the three cognitive function-related questions, were independently associated with the risk for needing LTC (hazard ratio: 1.40 for question 18; 1.18 for question 19; 1.22 for question 20), with highest hazard ratio found for question 18 (“Do your family or your friends point out your memory loss?”).

**Table 2:** Multivariate analysis of certification of need for LTC

Question no. or other item	LTC need		LTC need with dementia^a^	
	Hazard ratio	*P*	Hazard ratio	*P*
Age	1.15	< 0.001	1.19	< 0.001
Female sex	0.76	< 0.001	0.89	< 0.05
1	1.55	< 0.001	1.39	< 0.001
2	1.30	< 0.01	1.42	< 0.001
3	1.30	< 0.001	1.41	< 0.001
4	1.22	< 0.001	1.29	< 0.001
5	–^b^	–	1.17	< 0.05
6	1.43	< 0.001	1.39	< 0.001
7	1.09	0.138	–	–
8	1.14	< 0.05	–	–
9	1.16	< 0.01	1.24	< 0.001
10	1.14	< 0.01	1.11	< 0.05
11	1.42	< 0.001	1.40	< 0.001
12	1.65	< 0.001	1.62	< 0.001
13	–	–	–	–
14	0.77	< 0.001	0.67	< 0.001
15	0.90	< 0.05	0.82	< 0.001
16	1.34	< 0.001	1.34	< 0.001
17	1.25	< 0.001	1.19	< 0.01
18	1.40	< 0.001	1.73	< 0.001
19	1.18	< 0.01	1.19	< 0.01
20	1.22	< 0.001	1.35	< 0.001
21	1.14	< 0.05	1.19	< 0.01
22	–	–	1.12	0.108
23	–	–	–	–
24	–	–	–	–
25	1.17	< 0.001	1.11	0.066

^a^This category includes those whose need for LTC accompanied by dementia-related impairment in activities of daily living at grade IIa or higher

^b^Dashes indicate variables excluded from the model using Akaike’s information criterion

When analysis was confined to the “LTC need with dementia” group, a similar pattern was found, with all three cognitive function-related questions being significant predictors for LTC need (hazard ratio: 1.73 for question 18; 1.19 for question 19; 1.35 for question 20). Among the 25 questions in the KCL, the highest hazard ratio was found for question 18. Also, the hazard ratios associated with such questions were slightly higher for “LTC need with dementia”, compared to the whole LTC need.

## Discussion

This study used administrative data collected through the civil service and included responses from more than 50,000 older adults living in an urban area of Japan to demonstrate that asking cognitive function-related simple questions about ADL can identify people at risk of needing LTC.

The KCL questionnaire was devised to assess indicators of frailty, including cognitive decline; it was posted to nearly 80,000 citizens of Kobe aged ≥ 70 years in 2015, and the response rate was 64.4%. This response rate appears to be high for such large-scale surveillance, especially given that no reminders were sent. The average age of respondents, the ratio of men to women and the response rate (Table 1) were similar to those in our prior study [8], which assessed responses to the same questionnaire delivered from 2008 to 2010. However, the overall percentages of negative answers (indicating physical or mental difficulties) were generally lower in this study, perhaps owing to the government efforts to improve health literacy that began in 2006 [7]. In line with such trends, the percentages of negative answers to the three questions in the cognitive domain also tended to be lower [8]. Notably, the percentage of negative answers to the question “Do your family or your friends point out your memory loss?” (question 18) was 9.5% in this study, whereas it was 16.7% in our prior study, showing an apparent decrease occurring during the interval. According to the “Comprehensive survey of living conditions 2019” [3], the proportion of individuals aged ≥ 65 years who are living alone has substantially increased, and communication has become increasingly digitally recorded, both of which may have contributed to the decreased frequency of negative answers to question 18. Additionally, among the 25 questions, the average proportion of missing data was 1.7% (ranged from 0.5 to 6.9%), which is reasonably low for a large surveillance of this kind. Of note, because our prior study [8] was conducted in the same settings as this study, the rates of missing data could have been similarly lower, even though no information was available, potentially supporting the validity of the prior study findings.

Of note, the older adults who did not return the KCL showed a higher incidence of need for LTC than did respondents (12.5% vs 8.4%), representing a risk that was 1.5 times higher for those who did not respond. Also, such differences were consistent for each year during the 4-year follow-up (Fig. 1); this is in line with Igarashi et al. [10], and could be attributable to lower health literacy or potentially a higher prevalence of underlying illness in nonrespondents, and it requires further investigation. However, these findings imply that the inability to properly respond to such surveillance per se may be associated with a risk for needing LTC, suggesting the necessity of offering additional administrative help to this group. Nonetheless, because assessing such a large number of people is virtually impossible, improving health literacy through proactive public engagement may be a feasible approach. Indeed, the incidence of certification for LTC need appeared to be lower in this study (4.5% over 3 years) than in our prior study (5.4% over 3 years) [8] and other earlier studies [11–13], and focusing of the national LTC insurance system on prevention [7] could contribute to reducing this need, even though the absolute number of individuals certified for LTC has been increasing in proportion to the rapid ageing of society.

During the study period, the incidence of LTC need progressively increased as the number of negative answers in the cognitive domain increased (Fig. 2). In fact, 4 years’ cumulative incidence of LTC need in individuals who had three negative answers in this domain was six times higher (30.2%) than in those who had no negative answers (5.0%). Notably, among the respondents who had no negative answers in this domain, the risk of needing LTC was even lower than it was among the overall group of respondents (6.7%), suggesting that cognitive health is associated with a lower risk for LTC. These findings strengthen those of our prior study [8], which retrospectively assessed merged data from different sources and in which questions that were unanswered were considered to be negative responses for administrative purposes. When the need for LTC was confined to those certified to be with dementia, the incidence also increased as the number of negative answers increased to the questions in the cognitive domain (Fig. 3). Nonetheless, the incidence was only slightly lower than that found for the whole LTC need, which was not surprising because of the high prevalence of dementia in individuals with LTC need certification regardless of the causes. Indeed, two thirds (67%) of individuals with LTC need were with dementia, which may have mitigated the difference between the two analyses.

When responses were assessed by multivariate Cox regression analysis, many items in the KCL, including the three questions on cognitive function, were associated with the risk for LTC need (Table 2), This finding is concordant with other studies from our nation [12–14], which showed a value of KCL surveillance for the risk assessment of future LTC need, by utilizing administrative data of the local government. However, we are unaware of large-scale studies that prospectively evaluated how asking simple questions on cognitive function can be used to stratify the risk for needing LTC in the general population. Additionally, the hazard ratios associated with respective questions were roughly similar between the current study and our prior study [8], supporting the validity of our prior findings.

When the analysis was confined to those in the category of “LTC need with dementia”, all three questions in the cognitive domain remained significant predictors of LTC need. Of note, of all 25 questions, the highest hazard ratio (1.73) was found for the question about forgetfulness and repetition (question 18), suggesting its strong link with the future “LTC need with dementia”. Additionally, the hazard ratios associated with respective questions were only slightly higher for “LTC need with dementia”, compared to the whole LTC need (Table 2). This may be also explicable on the basis of high prevalence of dementia in individuals with LTC need of any causes. As the prevalence of dementia increases with the rapid ageing of society, and in the absence of effective therapeutic strategies to treat dementia, these findings provide a rationale for utilizing questionnaires to identify high-risk individuals who may receive the greatest benefit from a medical or administrative intervention.

Additionally, the approach adopted in this study may be applicable to municipalities in other countries because of its low cost. Incorporating such a questionnaire into local government activities will help to create a robust social system in which screening and risk assessment for dementia are routinely and simply implemented. In this way, appropriate services can be provided depending on each individual’s risk, the effects of which can be quantitatively evaluated in relation to the need for LTC. Thus, the current study may have established a model that can be used to collect quantitative feedback through local governmental services, and the information collected can lead to the reduction of societal burden of dementia.

However, this study has certain limitations. First, the causal relationships between negative answers in the cognitive domain and the need for LTC still remain to be addressed. Indeed, while cognitive decline is likely to predispose individuals to need LTC, having a condition that leaves an individual bedridden, for example, can accelerate cognitive decline, consequently leading to dementia. Also, dementia could be a concomitant manifestation of overall frailty among bedridden individuals who need LTC, which obscures the causal link between negative answers in the cognitive domain and LTC need. Second, the KCL survey was delivered and returned by post, but it may be conducted by more sophisticated electronic methods in the future. Third, although question 18 is primarily meaningful when an individual is living with someone, the proportion of individuals living alone is increasing [3], requiring optimization of the questions to meet lifestyle changes in society. Thus, continual improvements, as well as customization of questions to the target population, are necessary before extrapolating the findings of this study.

## Conclusion

The use of cognitive function-related simple questions, delivered through existing municipal administrative services, may help identify citizens at risk for LTC need and LTC need with dementia, and this low-cost approach could be used in various settings, suggesting its potential utility to explore new administrative approaches aimed at reducing the societal burden of LTC and dementia.

## Supplementary Information

**Additional file 1.** Daily life independence level with dementia. ADL impairment due to dementia is evaluated and categorized, ranging from independent to grade IV (worst impairment) in Japan. This definition is provided by the Ministry of Health, Labour and Welfare, Japan. Also available from: https://www.mhlw.go.jp/english/database/db-hss/dl/siel-2010-04.pdf.

## Data Availability

The original data set for this study was provided by Kobe city, under a written agreement between Kobe city, Kobe University and the Foundation for Biomedical Research and Innovation, in which data may not be provided to other parties.

## Abbreviations

ADL: Activities of daily living
KCL: Kihon Checklist
LTC: Long-term care
