# Supplementary material for: Implication of using cognitive function-related simple questions to stratify the risk of long-term care need: population-based prospective study in Kobe, Japan
Source: Health Res Policy Syst. 2022 Nov 29;20(Suppl 1):120. doi: 10.1186/s12961-022-00920-4 (PMC9706818; doi:10.1186/s12961-022-00920-4)
Supplement: Supplementary file 1 — Additional file 1. Daily life independence level with dementia. ADL impairment due to dementia is evaluated and categorized, ranging from independent to grade IV (worst impairment) in Japan. This definition is provided by the Ministry of Health, Labour and Welfare, Japan. Also available from: https://www.mhlw.go.jp/english/database/db-hss/dl/siel-2010-04.pdf. [file 12961_2022_920_MOESM1_ESM.pdf]

Grade	Definition
I	Has some sort of dementia but is almost independent in daily life in terms of domestic and social activities.
II	Symptoms, behaviours or difficulties in communicating that interfere with the person's daily life are observed to some degree, but is able to live independently with some help.<br>IIa: The condition described above occurring outside the home.<br>IIb: The condition described above occurring even at home.
III	Symptoms, behaviours or difficulties in communicating that interfere with the person's daily life are observed once in a while, and the person requires care.<br>IIIa: The condition described above occurring mainly during the daytime.<br>IIIb: The condition described above occurring mainly at night.
IV	Symptoms, behaviours or difficulties in communicating that interfere with the person's daily life are observed frequently, and the person requires constant care.

Source: adapted from Ministry of Health, Labour and Welfare, Japan. The Long-term care surveillance, [Internet]. Tokyo: Ministry of Health, Labour and Welfare; [cited 2022 Jan26]. Available from: https://www.mhlw.go.jp/english/database/db-hss/dl/siel-2010-04.pdf
